# Supplementary material for: Association between vitamin D status and circulating myokines (irisin, myostatin, and myonectin) in children: A cross-sectional study
Source: PLoS One. 2026 May 8;21(5):e0348873. doi: 10.1371/journal.pone.0348873 (PMC13155579; doi:10.1371/journal.pone.0348873)
Supplement: S1 File — (DOCX) [file pone.0348873.s001.docx]

| **S1 Table.** Robust regression models examining the effects of independent variables on circulating myokine levels in children with vitamin D deficiency. | | | | | | |
| --- | --- | --- | --- | --- | --- | --- |
| **Predictor** | **β_1_ (95% CI)** | **SE** | **β_2_** | **Test statistic** | ***p* value** | **VIF** |
| **Model 1: Dependent variable: Log(Irisin)** | | | | | | |
| Intercept | 0.679 (−0.145 to 1.504) | 0.401 | — | 1.693 | 0.102 | — |
| 25(OH)D (ng/mL) | −0.008 (−0.041 to 0.024) | 0.016 | −0.099 | −0.523 | 0.606 | 1.523 |
| Log(PTH) | 0.343 (0.050 to 0.636) | 0.142 | 0.489 | 2.408 | 0.023 | 1.764 |
| Age | −0.005 (−0.025 to 0.015) | 0.010 | −0.094 | −0.522 | 0.606 | 1.380 |
| Sex (female vs male) | −0.179 (−0.399 to 0.041) | 0.107 | −0.286 | −1.672 | 0.107 | 1.249 |
| BMI SDS | −0.093 (−0.191 to 0.006) | 0.048 | −0.357 | −1.936 | 0.064 | 1.453 |
| **Model 2: Dependent variable: Log(Myostatin)** | | | | | | |
| Intercept | 2.055 (1.195 to 2.916) | 0.418 | — | 4.912 | <0.001 | — |
| 25(OH)D (ng/mL) | −0.013 (−0.046 to 0.021) | 0.016 | −0.140 | −0.793 | 0.435 | 1.537 |
| Log(PTH) | 0.397 (0.093 to 0.701) | 0.148 | 0.513 | 2.681 | 0.013 | 1.798 |
| Age | −0.007 (−0.028 to 0.014) | 0.010 | −0.116 | −0.697 | 0.492 | 1.362 |
| Sex (female vs male) | −0.219 (−0.448 to 0.010) | 0.111 | −0.312 | −1.962 | 0.061 | 1.244 |
| BMI SDS | −0.110 (−0.211 to −0.009) | 0.049 | −0.388 | −2.237 | 0.034 | 1.474 |
| **Model 3: Dependent variable: Log(Myonectin)** | | | | | | |
| Intercept | −0.077 (−1.069 to 0.915) | 0.483 | — | −0.160 | 0.874 | — |
| 25(OH)D (ng/mL) | 0.000 (−0.039 to 0.039) | 0.019 | −0.0004 | −0.002 | 0.998 | 1.512 |
| Log(PTH) | 0.277 (−0.075 to 0.629) | 0.171 | 0.386 | 1.616 | 0.118 | 1.814 |
| Age | −0.003 (−0.027 to 0.022) | 0.012 | −0.050 | −0.239 | 0.813 | 1.377 |
| Sex (female vs male) | −0.129 (−0.392 to 0.134) | 0.128 | −0.198 | −1.007 | 0.323 | 1.225 |
| BMI SDS | −0.075 (−0.192 to 0.041) | 0.057 | −0.285 | −1.328 | 0.196 | 1.465 |
| **Notes:** β₁: Unstandardized regression coefficient, β₂: Standardized regression coefficient, CI: Confidence interval, VIF: Variance Inflation Factor, BMI SDS: Body mass index standard deviation score, 25(OH)D: 25-hydroxyvitamin D, PTH: Parathyroid hormone.  **Model summaries:**  In the robust regression model examining predictors of circulating irisin, the overall model was statistically significant (F = 3.354, *p* = 0.018; Durbin–Watson = 2.112; R² = 0.392). Log-transformed PTH was positively associated with Log(irisin) levels (β = 0.343, *p* = 0.023). Other variables were not significantly associated with irisin levels.  For myostatin, the regression model was also statistically significant (F = 4.625, *p* = 0.004; Durbin–Watson = 2.131; R² = 0.471). Log(PTH) showed a positive association with Log(myostatin) levels (β = 0.397, *p* = 0.013), whereas BMI SDS was negatively associated with myostatin concentrations (β = −0.110, *p* = 0.034). Other predictors were not statistically significant. For myonectin, the regression model did not reach statistical significance (F = 1.175, *p* = 0.348; Durbin–Watson = 1.867; R² = 0.184). | | | | | | |

| **S2 Table.** Multivariable linear regression models examining predictors of circulating myokine levels in the control group (outcome variables: irisin, myostatin, and myonectin). | | | | | | | | |
| --- | --- | --- | --- | --- | --- | --- | --- | --- |
|  | **β_1_ (95% CI)** | **SE** | **β_2_** | ***t* value** | ***p* value** | **Zero-order correlation** | **Partial correlation** | **VIF** |
| **Model 1: Outcome variable: Log(Irisin)** | | | | | | | | |
| Intercept | 1.829 (0.772 to 2.885) | 0.513 | — | 3.564 | 0.002 | — | — | — |
| 25(OH)D (ng/mL) | 0.015 (0.007 to 0.024) | 0.004 | 0.499 | 3.721 | 0.001 | 0.586 | 0.597 | 1.122 |
| Log(PTH) | -0.496 (-1.165 to 0.172) | 0.325 | -0.204 | -1.529 | 0.139 | -0.159 | -0.292 | 1.116 |
| Age | -0.049 (-0.075 to -0.022) | 0.013 | -0.491 | -3.76 | 0.001 | -0.572 | -0.601 | 1.068 |
| Sex (female vs male) | -0.015 (-0.252 to 0.221) | 0.115 | -0.018 | -0.134 | 0.894 | -0.019 | -0.027 | 1.085 |
| BMI SDS | -0.065 (-0.201 to 0.07) | 0.066 | -0.135 | -0.995 | 0.329 | 0.045 | -0.195 | 1.154 |
| **Model 2: Outcome variable: Log(Myostatin)** | | | | | | | | |
| Intercept | 3.698 (2.631 to 4.764) | 0.518 | — | 7.141 | <0.001 | — | — | — |
| 25(OH)D (ng/mL) | 0.012 (0.003 to 0.02) | 0.004 | 0.398 | 2.805 | 0.010 | 0.509 | 0.489 | 1.122 |
| Log(PTH) | -0.63 (-1.304 to 0.045) | 0.328 | -0.272 | -1.922 | 0.066 | -0.236 | -0.359 | 1.116 |
| Age | -0.048 (-0.075 to -0.021) | 0.013 | -0.513 | -3.706 | 0.001 | -0.569 | -0.595 | 1.068 |
| Sex (female vs male) | -0.014 (-0.252 to 0.225) | 0.116 | -0.017 | -0.119 | 0.906 | -0.004 | -0.024 | 1.085 |
| BMI SDS | -0.031 (-0.168 to 0.106) | 0.066 | -0.068 | -0.47 | 0.642 | 0.108 | -0.094 | 1.154 |
| **Model 3: Outcome variable: Log(Myonectin)** | | | | | | | | |
| Intercept | 1.085 (0.013 to 2.158) | 0.521 | — | 2.084 | 0.048 | — | — | — |
| 25(OH)D (ng/mL) | 0.016 (0.008 to 0.025) | 0.004 | 0.536 | 3.971 | 0.001 | 0.625 | 0.622 | 1.122 |
| Log(PTH) | -0.535 (-1.214 to 0.143) | 0.329 | -0.219 | -1.625 | 0.117 | -0.189 | -0.309 | 1.116 |
| Age | -0.043 (-0.07 to -0.016) | 0.013 | -0.433 | -3.294 | 0.003 | -0.521 | -0.550 | 1.068 |
| Sex (female vs male) | -0.037 (-0.277 to 0.203) | 0.116 | -0.042 | -0.315 | 0.755 | -0.058 | -0.063 | 1.085 |
| BMI SDS | -0.044 (-0.182 to 0.094) | 0.067 | -0.090 | -0.659 | 0.516 | 0.104 | -0.131 | 1.154 |
| **Notes:** β₁: Unstandardized regression coefficient, β₂: Standardized regression coefficient, CI: Confidence interval, VIF: Variance Inflation Factor,  BMI SDS: Body mass index standard deviation score, 25(OH)D: 25-hydroxyvitamin D, PTH: Parathyroid hormone.  **Model summaries:**  For irisin, the regression model was statistically significant (F = 7.501, *p* < 0.001; Durbin–Watson = 2.537; R² = 0.60; adjusted R² = 0.52). Serum 25(OH)D levels were positively associated with circulating irisin concentrations, whereas age showed a negative association. Other variables were not statistically significant.  For myostatin, the regression model was statistically significant (F = 6.146, *p* = 0.001; Durbin–Watson = 2.461; R² = 0.551; adjusted R² = 0.462). Serum 25(OH)D was positively associated with myostatin levels, while age showed a negative association.  For myonectin, the regression model was statistically significant (F = 7.340, *p* < 0.001; Durbin–Watson = 2.467; R² = 0.595; adjusted R² = 0.514). Serum 25(OH)D was positively associated with myonectin levels, whereas age showed a negative association. | | | | | | | | |
