# Supplementary material for: Association between vitamin D status and circulating myokines (irisin, myostatin, and myonectin) in children: A cross-sectional study
Source: PLoS One. 2026 May 8;21(5):e0348873. doi: 10.1371/journal.pone.0348873 (PMC13155579; doi:10.1371/journal.pone.0348873)
Supplement: Attachment — Submitted filename: REPORT-PONE-D-26-03690.pdf [file pone.0348873.s002.pdf]

Overview comments

I commend the authors of this Manuscript titled ***"Association between vitamin D status and circulating myokines (irisin, myostatin, and myonectin) in children: a cross-sectional study "***. This study explores a timely question on how vitamin D status relates to muscle-derived hormones (myokines) in children. However, I would encourage the authors to address a few gaps pointed in various sections to strengthen their work and contribute more robustly to the field.

Introduction

- ✓ The manuscript addresses a gap, but there is a need to expand the literature review by citing more recent systematic reviews and clarify why irisin, myostatin, and myonectin were chosen. Could you link the biological functions of irisin, myostatin, and myonectin to existing vitamin D pathways to provide a stronger theoretical framework.
- ✓ As much as the confounders like physical activity, nutrition and pubertal status are mentioned as limitations, there is a need for their deeper discussion in the introduction to strengthen the rationale and their potential impact in pediatric populations.

Methods

- ✓ Owing to the numerous biochemical variables in your study, it is important to assess multicollinearity among these variables to cater for correlation bias. I would advise you report correlation matrices or Variance Inflation Factor (VIF) if possible, in the supplementary material.

Results

- ✓ The main finding that children with vitamin D deficiency have lower levels of irisin, myostatin and myonectin is novel.
- ✓ The presentation of correlation analyses is well done, but without adjustment for multiple comparisons or confounders, the risk of false positives could arise. I suggest you apply a correction like Bonferroni for significant p-values to increase the robustness of the findings. If not possible to report correction for multiple comparisons, acknowledge the risk of type I error due to the high number of biochemical variables.

Discussion

- ✓ The discussion appropriately acknowledges the cross-sectional design's limitations and the need for longitudinal studies.
- ✓ Some limitations are acknowledged, but assay validation and confounder control need more emphasis. I propose that the authors consider expanding the discussion on how unmeasured confounders like physical activity and nutritional status might have influenced the observed myokine levels, even though they have been noted as limitations.

Conclusions

- ✓ They tie to the results but causal language could be avoided

Readability

- ✓ Line numbering is key in enhancing readability and ease of reference. This is missing
