# Supplementary material for: Association between vitamin D status and circulating myokines (irisin, myostatin, and myonectin) in children: A cross-sectional study
Source: PLoS One. 2026 May 8;21(5):e0348873. doi: 10.1371/journal.pone.0348873 (PMC13155579; doi:10.1371/journal.pone.0348873)
Supplement: Attachment — Submitted filename: Response_to_Reviewers.docx [file pone.0348873.s004.docx]

# **RESPONSE TO REVIEWER #1**

**Reviewer 1 – Comment 1**

*Rewrite / polish the Discussion (especially myostatin & limitations).*

**Response:**
We appreciate the reviewer's insightful feedback. The Discussion has been substantially revised to provide a more balanced interpretation of the myostatin findings. Specifically, we clarified the distinction between circulating myostatin levels and local tissue-level signaling, contextualizing these results within the broader concept of the bone–muscle unit. We also placed greater emphasis on the unique anabolic and developmental characteristics of pediatric physiology to avoid over-extrapolating the data. Finally, we removed speculative mechanistic statements and ensured that no causal inferences are drawn from our cross-sectional findings.

**Reviewer 1 – Comment 2**

*Add a strong Limitations paragraph (PLOS One–style).*

**Response:**
In line with PLOS ONE reporting standards, we have added a dedicated and comprehensive Limitations paragraph to the Discussion. We now explicitly note the constraints of our cross-sectional design and the absence of an a priori power calculation. We also discuss the potential impact of unmeasured confounders (such as physical activity, pubertal stage, and seasonal variations), the inherent analytical limitations of ELISA-based assays, and the lack of established pediatric reference ranges for circulating myokines. Lastly, we acknowledge that the lack of functional muscle assessments and the specific clinical profile of our cohort (predominantly symptomatic children with secondary hyperparathyroidism) may restrict the generalizability of our findings to milder forms of vitamin D deficiency.

**Reviewer 1 – Comment 3**

*Prepare a formal “Response to Reviewers” letter.*

**Response:**

A detailed, point-by-point response to all reviewer comments has been prepared and is submitted alongside the revised manuscript.

**Reviewer 1 – Comment 4**

*Language editing + scientific tone polishing for the whole manuscript.*

**Response:**

The entire manuscript has been carefully reviewed and edited to improve clarity, readability, and scientific tone. Minor linguistic refinements have been incorporated throughout the clean version of the text to ensure academic rigor and conciseness.

**Reviewer 1 – Comment 5**

*Improve the Abstract (results & conclusions).*

**Response:**
The Abstract has been revised to improve quantitative clarity and scientific precision. In the Results section, we added the specific median myokine concentrations, correlation coefficients (*r* values), and their corresponding *p* values to provide a clearer numerical context. Additionally, the Conclusions were adjusted to emphasize the associative nature of our findings, explicitly noting the cross-sectional design and avoiding any language that might imply causality.

RESPONSE TO REVIEWER #2

## **REVIEWER 2 –OVERVIEW COMMENTS** *I commend the authors of this Manuscript titled “Association between vitamin D status and circulating myokines (irisin, myostatin, and myonectin) in children: a cross-sectional study “. This study explores a timely question on how vitamin D status relates to muscle-derived hormones (myokines) in children. However, I would encourage the authors to address a few gaps pointed in various sections to strengthen their work and contribute more robustly to the field.*

## **Response:**

We thank the reviewer for the positive evaluation and for the helpful suggestions. The manuscript has been revised accordingly, and each point is addressed below.
**INTRODUCTION**

**Reviewer 2 – Comment**
*The manuscript addresses a gap, but there is a need to expand the literature review by citing more recent systematic reviews and clarify why irisin, myostatin, and myonectin were chosen. Could you link the biological functions of irisin, myostatin, and myonectin to existing vitamin D pathways to provide a stronger theoretical framework.*
**Response:**

The Introduction has been revised to provide a clearer theoretical framework. We added recent review literature on skeletal muscle endocrine function and myokine biology (Refs. 8, 13, 14). The rationale for focusing on irisin, myostatin, and myonectin is now described more explicitly, emphasizing their roles in muscle metabolism, growth regulation, and lipid–glucose homeostasis within the bone–muscle endocrine axis. In addition, we expanded the description of potential biological links between vitamin D signaling and muscle endocrine activity, including vitamin D receptor–mediated pathways and the possible influence of secondary hyperparathyroidism (Refs. 3, 6, 12, 15). We also clarified the knowledge gap, noting that although evidence is increasing in adult populations, pediatric data remain limited.

**Reviewer 2 – Comment**
*As much as the confounders like physical activity, nutrition and pubertal status are mentioned as limitations, there is a need for their deeper discussion in the introduction to strengthen the rationale and their potential impact in pediatric populations.*

**Response:**
We revised the Introduction to include a short paragraph describing developmental and environmental factors that may influence myokine secretion in children. This section now highlights the potential roles of physical activity, nutritional status, and pubertal maturation in regulating muscle-derived endocrine signaling (Refs. 14, 16, 17). These additions clarify the rationale for examining vitamin D–related changes in circulating myokines within the context of growth and development.

**METHODS**

**Reviewer 2 –Comment**
*Owing to the numerous biochemical variables in your study, it is important to assess multicollinearity among these variables to cater for correlation bias. I would advise you report correlation matrices or Variance Inflation Factor (VIF) if possible, in the supplementary material.*
**Response:**

We thank the reviewer for this important and constructive methodological suggestion. We agree that assessing multicollinearity is essential given the number of biochemical variables included in our regression models.

To address this concern, we calculated the Variance Inflation Factor (VIF) for all independent variables included in the multivariable regression models. As suggested, the regression models together with their corresponding VIF values are now provided as supplementary material **(S1 Table and S2 Table)**.

All calculated VIF values were below the conservative threshold of 5, indicating that there is no problematic multicollinearity among the independent variables. A clarifying statement regarding the VIF assessment has also been added to the Statistical Analysis section of the revised manuscript.

**RESULTS**

**Reviewer 2 –Comment**

*The main finding that children with vitamin D deficiency have lower levels of irisin, myostatin and myonectin is novel.*
*The presentation of correlation analyses is well done, but without adjustment for multiple comparisons or confounders, the risk of false positives could arise. I suggest you apply a correction like Bonferroni for significant p-values to increase the robustness of the findings. If not possible to report correction for multiple comparisons, acknowledge the risk of type I error due to the high number of biochemical variables.*

**Response:**

We sincerely thank the reviewer for this highly constructive and critical feedback. We completely agree that the high number of biochemical variables in our correlation matrix increases the risk of Type I error (false positives). To address this critical concern and increase the robustness of our findings, we have completely revised our correlation analysis.

First, instead of a pooled analysis, we now present the correlation matrix separately for the Vitamin D-deficient and Control groups to provide a clearer physiological context (**Revised Table 3**). Furthermore, all *p*-values in this updated analysis have been rigorously adjusted for multiple comparisons using the Benjamini-Hochberg False Discovery Rate (FDR) method. We preferred the FDR approach over the Bonferroni correction, as FDR effectively controls false positives while maintaining statistical power for detecting true associations in exploratory biochemical studies. The Statistical Methods, Results, and Discussion sections have been revised accordingly to reflect these adjusted values.

## **DISCUSSION**

## **Reviewer 2 –Comment**

*The discussion appropriately acknowledges the cross-sectional design’s limitations and the need for longitudinal studies.*

**Response:**

We appreciate the reviewer’s recognition of our effort to maintain a balanced and cautious interpretation of the cross-sectional data.

**Reviewer 2 –Comment**

## *Some limitations are acknowledged, but assay validation and confounder control need more emphasis. I propose that the authors consider expanding the discussion on how unmeasured confounders like physical activity and nutritional status might have influenced the observed myokine levels, even though they have been noted as limitations.*

**Response:**

We agree with the reviewer that these unmeasured confounders warrant deeper consideration. Accordingly, we have expanded the Discussion to address how biological and contextual factors might influence circulating myokine levels. As suggested, we added a dedicated paragraph examining the roles of physical activity, nutritional status (including energy balance and protein intake), and pubertal maturation as significant modulators of muscle endocrine signaling (Refs. 14–17). Furthermore, we elaborated on the methodological caveats of using ELISA kits for myokine quantification, specifically highlighting the absence of established pediatric reference ranges (Ref. 36). We believe these revisions provide a much more balanced interpretation, clearly framing our findings as relative associations within this cohort rather than definitive indicators of muscle dysfunction.

## **CONCLUSIONS**

## **Reviewer 2 –Comment**

*They tie to the results but causal language could be avoided.*

**Response:**

The Conclusions section has been revised to ensure that the findings are described using associative language and do not imply causality.

**READABILITY**

**Reviewer 2 -Comment**

*Line numbering is key in enhancing readability and ease of reference. This is missing.*

**Response:**

Line numbering has been added to the revised manuscript to improve readability and facilitate the review process.
